# Low Rates of Discontinuation Unrelated to Disease Progression with Trastuzumab Deruxtecan in Metastatic Breast Cancer: A Real-World Study

**DOI:** 10.3390/cancers18172882

**Published:** 2026-09-06

**Authors:** Luca Licata, Francesca Patanè, Alessandra Guarino, Barbara Galbardi, Annarita Battaglia, Marco Mariani, Caterina Zanibelli, Giulia Notini, Matteo Maria Naldini, Carlo Bosi, Antonella Chiavassa, Marta Piras, Irene Persano, Alessia Rognone, Lorenzo Sica, Stefania Zambelli, Patrizia Zucchinelli, Giulia Viale, Giampaolo Bianchini

**Affiliations:** 1 Department of Medical Oncology, IRCCS San Raffaele Scientific Institute, 20132 Milan, Italy; licata.luca@hsr.it (L.L.); guarino.alessandra@hsr.it (A.G.); galbardi.barbara@hsr.it (B.G.); battaglia.annarita@hsr.it (A.B.); mariani.marco@hsr.it (M.M.); zanibelli.caterina@hsr.it (C.Z.); notini.giulia@hsr.it (G.N.); naldini.matteomaria@hsr.it (M.M.N.); bosi.carlo@hsr.it (C.B.); persano.irene@hsr.it (I.P.); rognone.alessia@hsr.it (A.R.); sica.lorenzo@hsr.it (L.S.); zambelli.stefania@hsr.it (S.Z.); zucchinelli.patrizia@hsr.it (P.Z.); viale.giulia@hsr.it (G.V.); 2 Humanitas Cancer Center, IRCCS Humanitas Research Hospital, 20089 Rozzano, Italy; francesca.patane@cancercenter.humanitas.it; 3 School of Medicine and Surgery, Vita-Salute San Raffaele University, 20132 Milan, Italy; 4 Yale School of Medicine, Yale Cancer Center, New Haven, CT 06510, USA; 5 Medica Scientia Innovation Research (MEDSIR), 08005 Barcelona, Spain; 6 CRUK Cambridge Institute, University of Cambridge, Cambridge CB2 0RE, UK; 7 Department of Radiation Oncology, New York-Presbyterian/Weill Cornell Medicine, New York, NY 10065, USA; 8 Department of Medical Oncology, Santa Croce e Carle Hospital, 12100 Cuneo, Italy; chiavassa.a@ospedale.cuneo.it; 9 Division of Medical Senology, IEO European Institute of Oncology, IRCCS, 20141 Milan, Italy; marta.piras@ieo.it

**Keywords:** trastuzumab deruxtecan, metastatic breast cancer, treatment discontinuations, real-world

## Abstract

**Purpose**: Trastuzumab deruxtecan (T-DXd) has significantly improved outcomes in patients with HER2-positive and HER2-low/ultralow metastatic breast cancer (MBC). However, most patients eventually discontinue treatment. In clinical trials, discontinuation without disease progression occurred in 30–60% of cases, but real-world data on the reasons for discontinuation and subsequent treatment strategies remain limited. This study aimed to describe T-DXd discontinuation patterns and post-discontinuation treatments in a real-world setting. **Methods**: We conducted a single-center retrospective observational study including consecutive patients with MBC treated with T-DXd between July 2018 and December 2025. Patients who discontinued T-DXd for any reason were included. Reasons for discontinuation, treatment duration, response outcomes, and subsequent systemic therapies were analyzed using descriptive statistics. **Results**: Overall, 93 patients were included: 71.0% had HER2-positive, 26.9% HER2-low, and 2.1% HER2 0 tumors. The primary reason for discontinuation was progressive disease (82.8%). Other reasons included adverse events (14.0%), non-treatment-related causes (2.2%) and patient decision (1.1%). Interstitial lung disease represented the most common toxicity leading to discontinuation (10.8%). The median treatment duration was 7.9 months overall and was longer in patients with HER2-positive than in thosewith HER2-low and HER2 0 disease. Among evaluable patients, overall response rate was 83.1% in HER2-positive tumors and 52% in HER2-low tumors. After discontinuation, 80.6% of patients received subsequent therapy. Patients discontinuing for reasons other than progression had longer treatment exposure. **Conclusions**: In this real-world cohort, most T-DXd discontinuations were due to disease progression, while adverse event-related discontinuations were less frequent than in clinical trials. Several factors may influence treatment persistence, including toxicity management. Further evaluation in larger, multicenter cohorts is warranted to better characterize their contribution.

## Simple Summary

Trastuzumab deruxtecan is an effective treatment for patients with metastatic breast cancer. However, treatment may sometimes need to be permanently discontinued because of side effects before disease progression. In clinical trials, discontinuations for reasons other than disease progression have been relatively frequent, with side effects being the most common cause. We conducted a retrospective study including 93 patients with metastatic breast cancer who permanently discontinued trastuzumab deruxtecan at our institution, to better understand why and when treatment was stopped and which treatments were given afterwards. Most patients (83%) discontinued treatment because their disease progressed, while 14% stopped because of side effects. This rate was lower than that reported in clinical trials. This difference may be related to differences in patient populations and treatment settings, as well as to clinical experience and toxicity management in specialized centers. Larger, multicenter studies are needed to determine whether these factors contribute to longer treatment persistence in routine practice.

## 1. Introduction

Trastuzumab deruxtecan (T-DXd) is an antibody-drug conjugate (ADC) consisting of an anti-human epidermal growth factor receptor 2 (HER2) antibody linked to the topoisomerase I inhibitor deruxtecan, a camptothecin derivative, via a protease-cleavable tetrapeptide linker [1].

This ADC has reshaped the treatment of metastatic breast cancer (MBC), demonstrating a substantial clinical benefit in patients with both HER2-positive (HER2+) and HER2-low/ultralow tumors, outperforming the previous standard of care treatments in these settings [2,3,4]. However, most patients with MBC receiving T-DXd ultimately discontinue the drug due to disease progression, toxicity, or other reasons. A significant proportion of these patients are eligible for additional anti-cancer therapies.

Given the high efficacy of T-DXd and the resulting prolonged duration of treatment in many patients, discontinuation without disease progression has emerged as a clinically relevant issue, ranging from about 30% to over 60% in clinical trials [5,6,7,8,9,10], with rates varying widely depending on patient population, line of therapy, and follow-up duration. Among reasons for discontinuation other than progressive disease, adverse events are the most frequent, accounting for approximately 18% to nearly 35% of overall discontinuations [5,6,7,8,9,10]. In particular, interstitial lung disease (ILD)/pneumonitis, a known risk of T-DXd, led to discontinuation of the drug in about 8–12% of patients [5,6,7,8,9,10]. Other less common reasons include patient decision (ranging between roughly 3–11%, with rates increasing with longer treatment duration), physician’s decision or death.

Premature treatment discontinuations in real-world practice may differ from those observed in controlled trial settings and may be influenced by multiple factors, including differences in institutional experience, patient population, physician familiarity with T-DXd toxicity management, and patient counseling strategies, with a potential detrimental impact on overall therapeutic benefit.

Although several real-world studies have confirmed the effectiveness and safety of T-DXd across different clinical settings, they have primarily focused on response, survival, and overall safety outcomes. In contrast, detailed characterization of treatment discontinuation patterns, including the specific reasons for permanent discontinuation, dose modifications, and subsequent treatment trajectories, remains limited.

In this study, we therefore aimed to characterize the reasons and timing of T-DXd discontinuation in routine clinical practice and to describe post-discontinuation treatment approaches, providing a focused assessment of the clinical trajectory of patients after permanent treatment cessation.

## 2. Methods

### 2.1. Study Design, Participants and Data Sources

This single-center, retrospective observational study was conducted at IRCCS San Raffaele Scientific Institute, Milan (Italy) between September 2025 and February 2026. Clinicopathologic and demographic data from consecutive patients with MBC who received at least one dose of T-DXd, administered in any line of therapy between July 2018 and December 2025, were retrospectively collected from medical health records. Only patients who permanently discontinued T-DXd for any reason were included in the study. Patients were categorized as having HER2+ MBC if at least one biopsy performed prior to T-DXd initiation showed HER2 immunohistochemistry (IHC) 3+ or IHC 2+ with amplified in situ hybridization (ISH). In the absence of any HER2+ biopsy, tumors were classified as HER2-low if at least one biopsy showed IHC 1+ or IHC 2+ without ISH amplification, or as HER2 0 if all available biopsies showed IHC 0.

### 2.2. Objectives and Outcome Measures

The primary objective of the study was to describe reasons and timing of T-DXd discontinuation in a real-world population of patients with MBC. The primary outcome measure of the study was the rate and reason of treatment discontinuation.

Reasons for treatment discontinuation were categorized as progressive disease (clinical or radiological, including death due to disease progression), adverse events (including death related to toxicity), patient decision, or other causes based on the treating physician’s documentation.

Secondary objectives included the evaluation of T-DXd activity and exposure, and the description of post-discontinuation therapies. Outcome measures of activity included the overall response rate (ORR), defined as the proportion of patients achieving complete or partial response; and disease control rate (DCR), defined as the proportion of patients achieving complete response, partial response or stable disease. Treatment exposure was evaluated using duration of treatment (DoT), defined as the time from T-DXd initiation to permanent discontinuation for any cause. Post-progression therapy evaluation included the description of the first subsequent systemic anticancer treatment initiated following T-DXd discontinuation and its corresponding duration.

### 2.3. Assessments

Baseline disease evaluation was carried out using total-body computed tomography (CT) in all patients. Tumor response was assessed using imaging performed according to institutional clinical practice. Radiological response was locally evaluated according to RECIST version 1.1 criteria [11]. All patients underwent high-resolution chest CT scans every 6–9 weeks for ILD monitoring. Additional imaging studies, including bone scan, brain MRI, and PET, were performed as clinically indicated.

Supportive care, including antiemetic prophylaxis, ILD surveillance, and dose modifications, was delivered according to institutional clinical practice throughout treatment.

### 2.4. Safety Evaluation

Adverse events were retrospectively extracted from medical records and categorized according to the treating physician’s documentation. Events were graded according to the Common Terminology Criteria for Adverse Events (CTCAE), version 5.0.

Rates and reasons for T-DXd dose reductions were also recorded.

### 2.5. Statistics

Descriptive statistics were used to summarize patient and tumor characteristics. Continuous variables were expressed as the median or mean and range. Categorical variables are expressed as numbers and proportions (%). Statistical analyses were performed using R environment, version 4.5.2 (http://www.r-project.org).

### 2.6. Ethics and Oversight

This retrospective study analyzed anonymized data from previously collected patient information and did not involve any intervention or impact on patient care. All patients had provided informed consent before undergoing treatment with T-DXd. Patients were included in an observational study primarily aimed at describing the real-world management of T-DXd-related nausea and vomiting (“TD-rNV”) [12]. The study protocol was approved by the institutional Ethics Committee and followed local regulation and ethical guidelines.

## 3. Results

As of December 2025, a total of 110 patients had received T-DXd at our institution, of whom 93 patients permanently discontinued treatment and were included in the present analysis. Among them, 71.0% (*n* = 66) had HER2+, 26.9% (*n* = 25) HER2-low, and 2.1% (*n* = 2) HER2 0 tumors. All patients were female, with a mean age of 57.1 years (range 24–85). T-DXd was administered as first-line therapy in 5.4% of patients (*n* = 5), second-line in 18.3% (*n* = 17), third-line in 31.2% (*n* = 29), and ≥fourth-line in 45.1% (*n* = 42). Visceral metastases were present in 75.3% (*n* = 70), while 30.1% (*n* = 28) had brain metastases. Baseline patient characteristics in the overall population and according to HER2 status are summarized in Table 1.

The primary reason for T-DXd discontinuation was progressive disease (82.8%, *n* = 77). Consequently, discontinuations unrelated to disease progression accounted for a relatively small proportion of cases. Other reasons included adverse events (14.0%, *n* = 13) and patient decision (1.1%, *n* = 1). Two patients discontinued T-DXd due to non-treatment-related causes (COVID-19 pneumonia and polytrauma).

**Figure 1:**
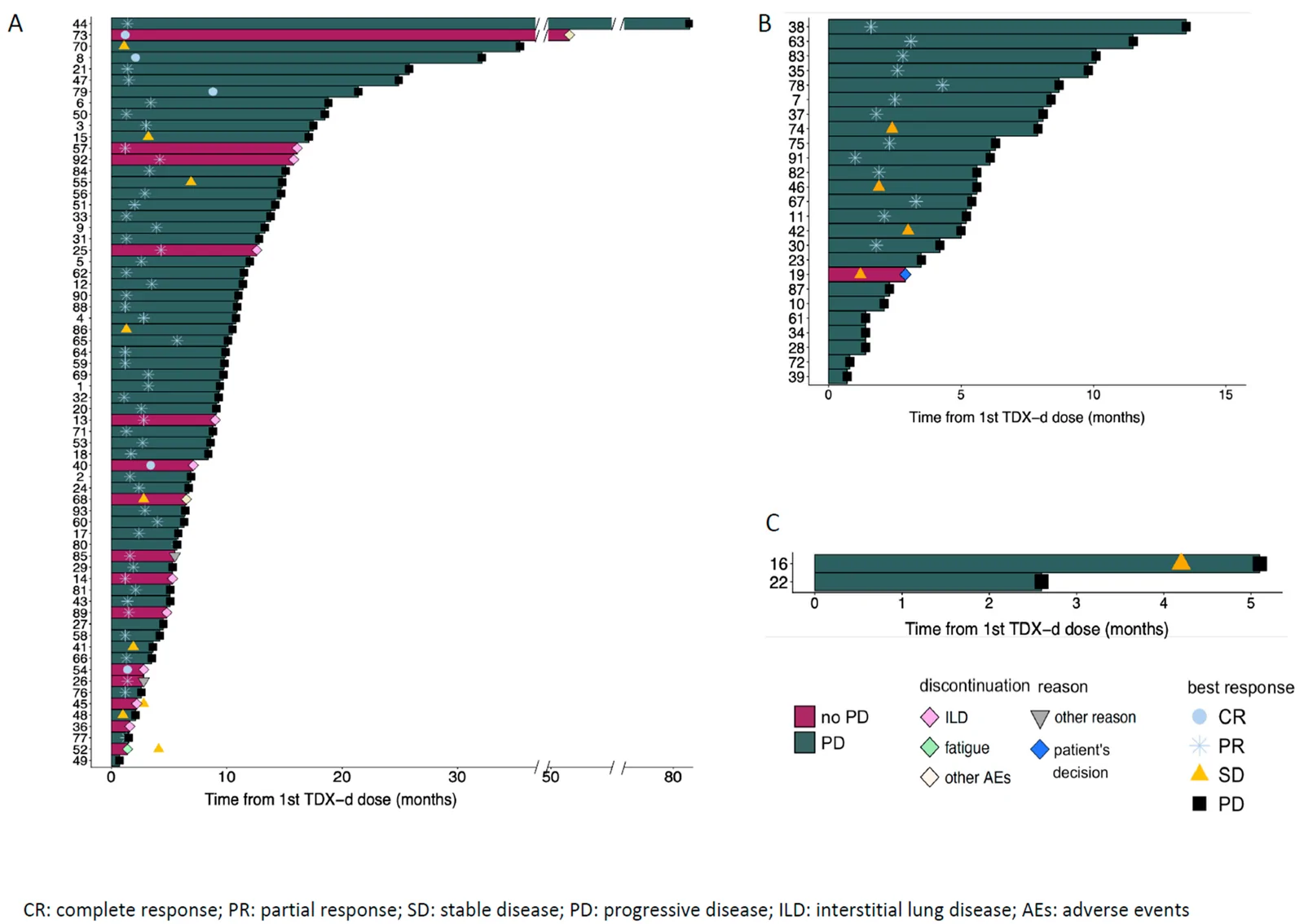
Swimmer plot showing individual patient data of treatment duration, discontinuation reasons and best response of patients treated with trastuzumab deruxtecan. (**A**) HER2+ (*n* = 66). (**B**) HER2-low (*n* = 25). (**C**) HER2 0 (*n* = 2).

Among the 66 patients with HER2+ tumors, 77.3% (*n* = 51) and 19.7% (*n* = 13) discontinued T-DXd due to progressive disease and adverse events, respectively (Figure 1).

All patients with HER2-low tumors except one (who discontinued by personal choice) as well as all patients with HER2 0 tumors discontinued T-DXd due to progressive disease (Figure 1).

Adverse events leading to treatment discontinuation included ILD in 10.8% of patients (*n* = 10), as well as fatigue (*n* = 1), increased liver function tests (*n* = 1), and persistent neutropenia (*n* = 1).

Among the 10 ILD cases, four were Grade 1 (not resolved to Grade 0), one Grade 2, two Grade 3, and three Grade 5. The three Grade 5 ILD events were managed at local emergency departments outside our Institution for patients’ proximity. In one case, a concomitant opportunistic Aspergillus infection was documented.

One additional ILD case occurred among the 17 patients remaining on T-DXd, resulting in an overall ILD incidence of 10.0% (11/110) among patients treated at our center.

Prior to permanent discontinuation, 21.5% of patients (*n* = 20) required ≥1 dose reduction, most frequently due to fatigue (9.7%, *n* = 9), increased liver function tests (6.4%, *n* = 6), and nausea (3.2%, *n* = 3).

The median DoT was 7.9 months (range, 0.7–81.4) in the overall population. Median DoT was longer in patients with HER2-positive tumors (9.4 months; range, 0.7–81.4), compared with those with HER2-low (5.4 months; range, 0.7–13.5) and HER2 0 disease (3.9 months; range, 2.6–5.1) (Figure 1).

**Figure 2:**
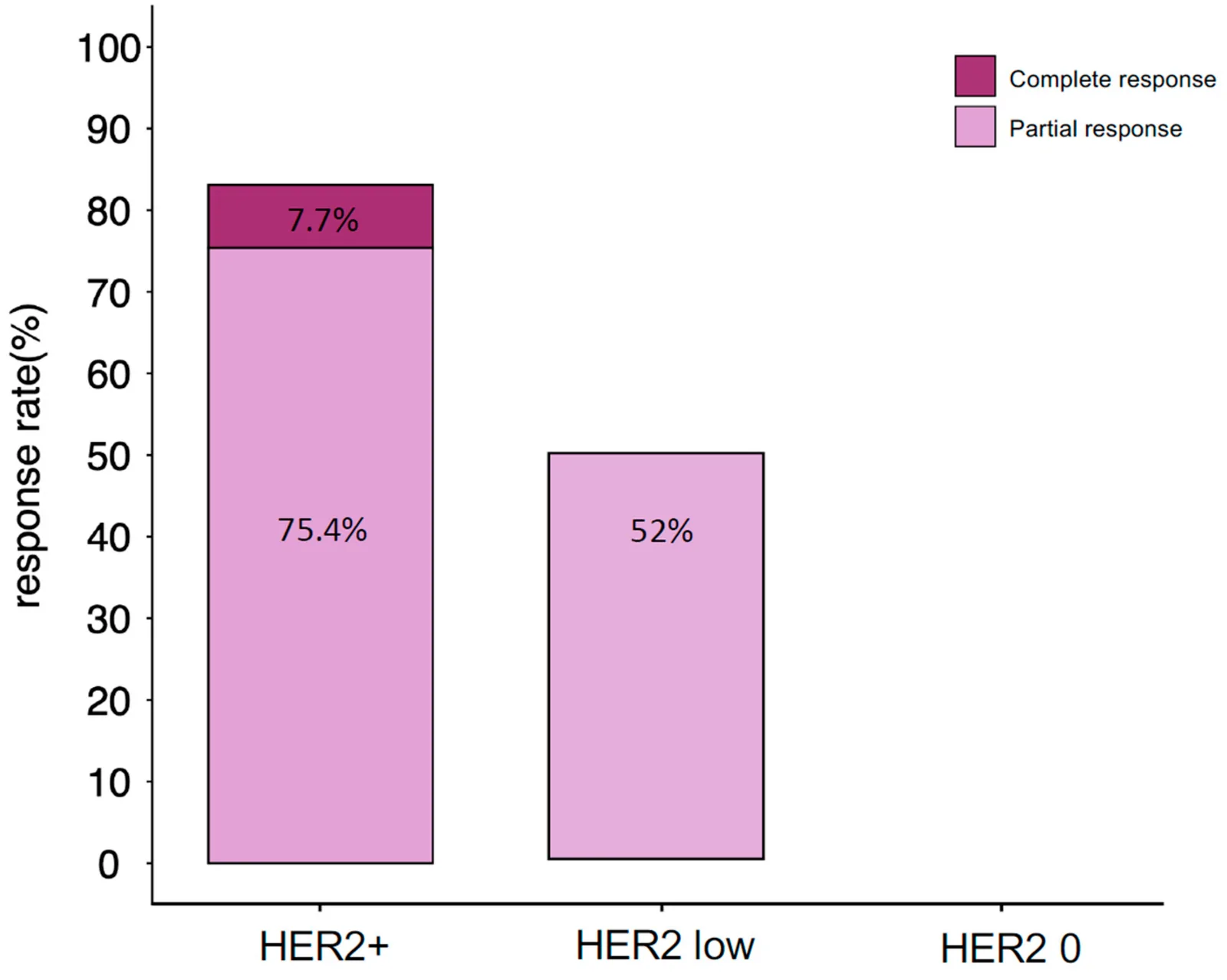
Objective response rate by tumor subtype.

Among patients with HER2+ tumors evaluable for response (*n* = 65/66), the ORR was 83.1%, including 7.7% complete responses and 75.4% partial responses (Figure 2). The DCR was 96.9%, with stable disease observed in 13.8% of patients. Progressive disease as best response was reported in two patients, both receiving T-DXd in very late therapy lines (10th and 14th line for metastatic disease, respectively).

Among evaluable patients with HER2-low tumors (*n* = 25/25), the ORR was 52%, consisting exclusively of partial responses (*n* = 13) (Figure 2). The DCR was 68%. Progressive disease as best response was observed in 32% of patients (*n* = 8); among these patients, one received T-DXd as third line, one as fifth line, and six in the sixth-line setting or beyond.

No objective responses were observed in the HER2 0 subgroup: one patient achieved stable disease when treated in the third-line setting, whereas the other experienced progressive disease as best response in the sixth-line setting.

Overall, 80.6% of patients (*n* = 75) received a subsequent line of therapy following T-DXd discontinuation. Among patients with HER2+ tumors who received subsequent treatment (*n* = 53, 80.3% of the total), the most common regimen was trastuzumab plus tucatinib plus capecitabine (*n* = 20, 37.7%), followed by chemotherapy alone (*n* = 9, 17%), chemotherapy plus trastuzumab (*n* = 7, 13.2%), T-DM1 (*n* = 5, 9.4%), trastuzumab plus endocrine therapy (*n* = 5, 9.4%), chemotherapy plus other anti-HER2 therapy (*n* = 4, 7.6%), and trastuzumab maintenance (*n* = 3, 5.7%).

**Figure 3:**
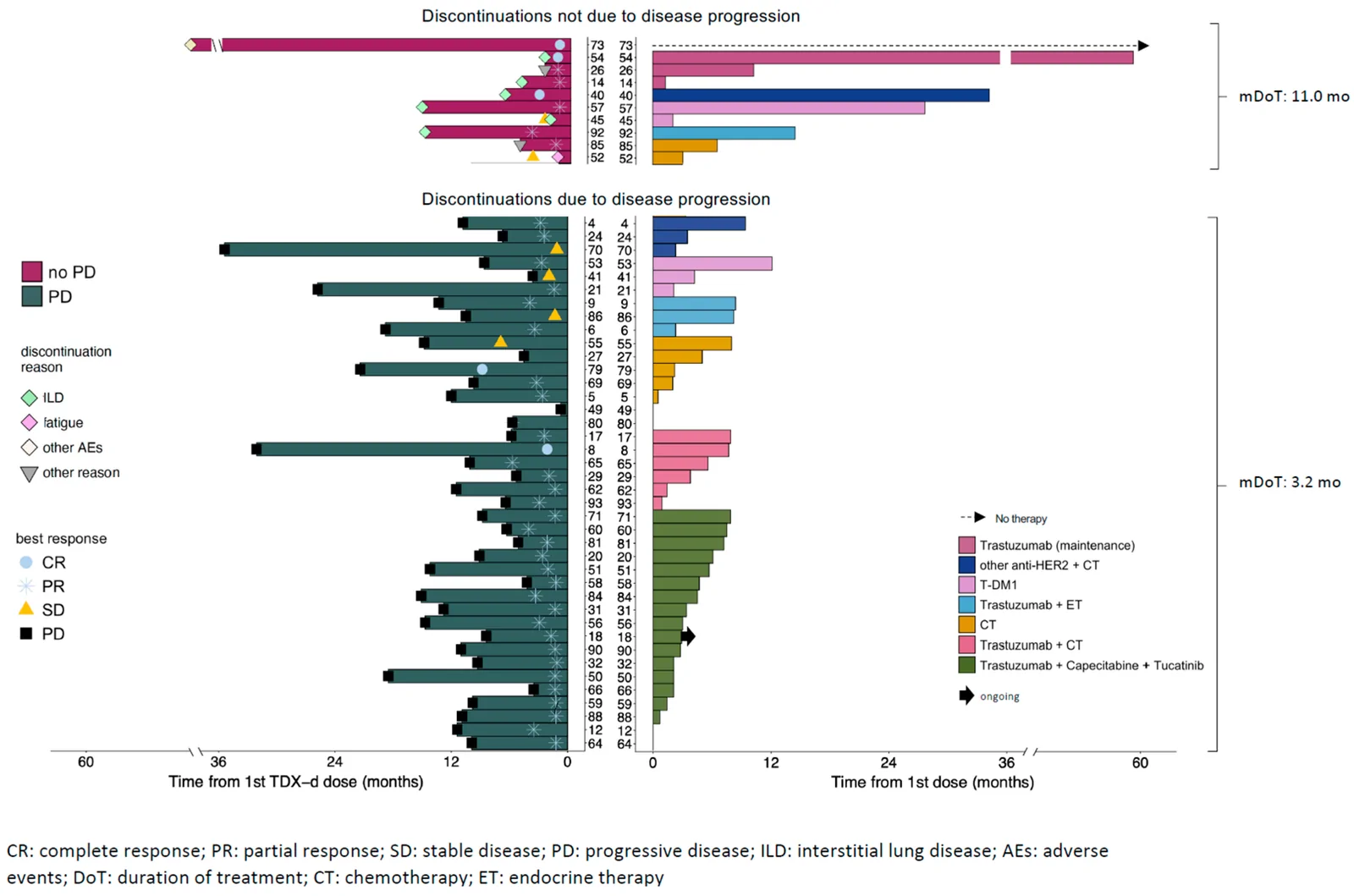
Swimmer plot showing individual patient data of type and duration of subsequent therapies in patients with HER2+ metastatic breast cancer who discontinued trastuzumab deruxtecan.

Median DoT for subsequent therapies ranged from 2.2 months of chemotherapy alone to 11.0 months for trastuzumab maintenance (Figure 3).

**Figure 4:**
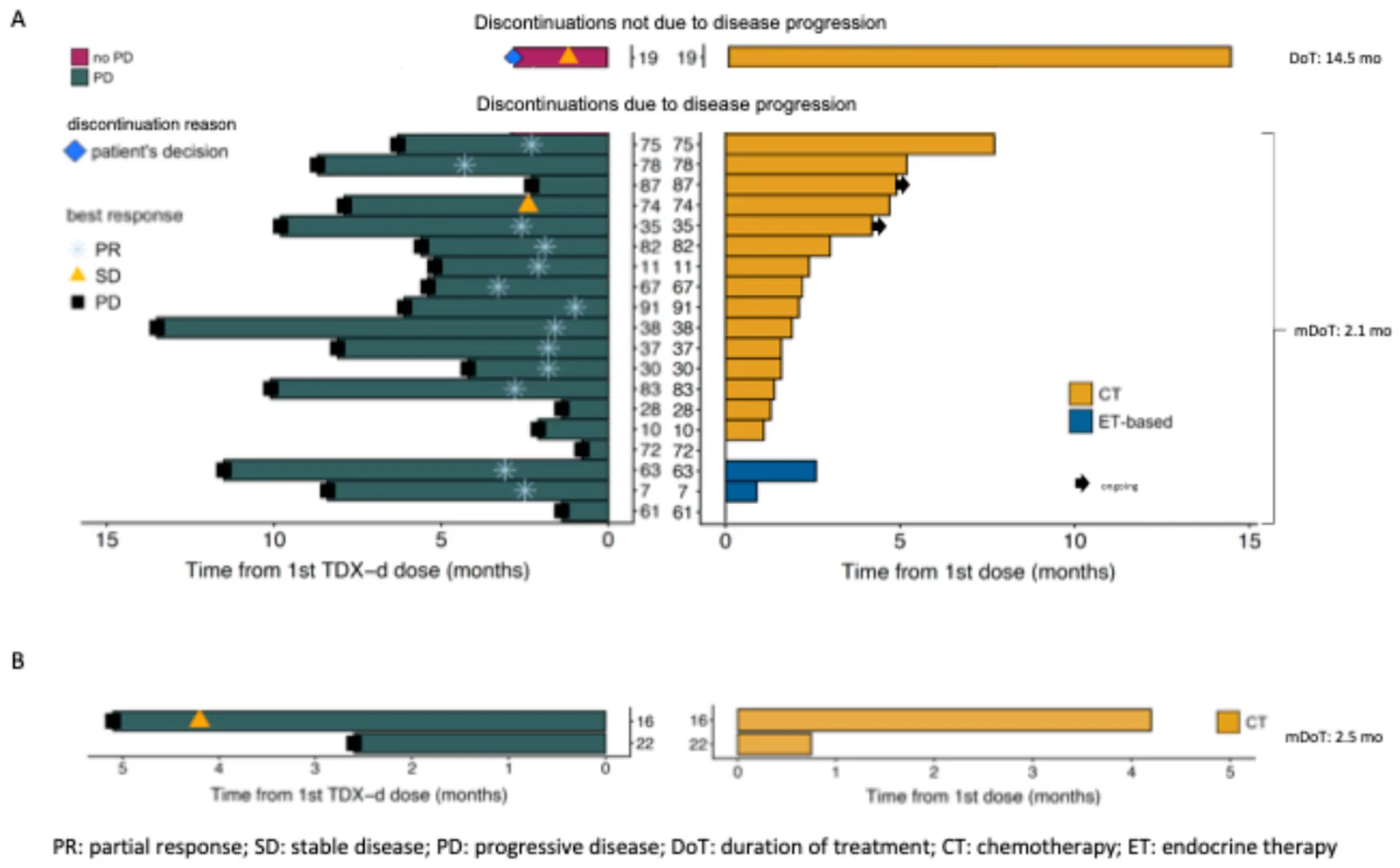
Swimmer plot showing individual patient data of type and duration of subsequent therapies in patients who discontinued trastuzumab deruxtecan. (**A**) HER2-low (*n* = 25). (**B**) HER2 0 (*n* = 2).

Among patients with HER2-low tumors receiving a subsequent line (*n* = 20, 88% of the total), 85% (*n* = 17) received chemotherapy and 15% (*n* = 3) endocrine therapy, with a median DoT of 2.2 and 1.0 months, respectively (Figure 4).

Both patients with HER2 0 tumors received chemotherapy following T-DXd discontinuation. The median DoT was 2.4 months (Figure 4).

Notably, patients who discontinued treatment for reasons other than disease progression had a numerically longer median DoT than those who discontinued due to progressive disease (12.7 vs. 2.6 months in the overall population). Among patients with HER2+ tumors, median DoT was 11.0 months in those without progression and 3.4 months in those with progressive disease on T-DXd (Figure 3). The single patient with HER2-low disease who discontinued for reasons other than progression had a DoT of 14.5 months, compared with a median DoT of 2.1 months among those who discontinued due to progressive disease (Figure 4).

Among the 18 patients who did not receive subsequent therapy, nine died while on T-DXd: five due to progressive disease, three due to adverse events (ILD), and one due to unrelated liver cirrhosis.

Five patients were unable to receive further treatment due to clinical deterioration, while three patients were lost to follow-up after referral to external institutions. One patient with HER2+ disease, who achieved a complete response at the first disease assessment, discontinued T-DXd in January 2024 due to persistent neutropenia and has since remained under follow-up without receiving further systemic therapy.

## 4. Discussion

In this real-world cohort of patients with metastatic breast cancer treated with T-DXd, most permanent discontinuations were driven by disease progression (83%), while only 17% of patients discontinued treatment for reasons unrelated to progression. 

This proportion appears lower than that reported in pivotal clinical trials leading to T-DXd approval. These findings are clinically relevant, as premature discontinuation of highly active therapies such as T-DXd may limit the overall therapeutic benefit achievable in metastatic breast cancer. In pivotal studies evaluating T-DXd in HER2-positive and HER2-low metastatic breast cancer, discontinuation rates unrelated to progressive disease varied considerably, ranging from approximately 30% to over 60% (Supplementary Table S1), depending on study design, follow-up duration, and treatment line [5,6,7,8,9,10].

Several factors may have contributed to this apparent discrepancy, although their relative contribution cannot be determined from this retrospective observational analysis.

Clinical trials often report longer treatment exposure, which may increase the probability of cumulative toxicities or elective treatment discontinuation, particularly in patients experiencing prolonged disease control. However, comparisons of treatment duration between our cohort and clinical trials should be interpreted with caution, as treatment duration in our series was calculated only among patients who discontinued therapy.

It is also possible that the shorter treatment duration observed in our cohort reflects less favourable baseline prognostic characteristics, including the relatively high prevalence of brain metastases (30% of patients) and the heavily pretreated population (45% receiving T-DXd in ≥4th line). The retrospective design of the study precludes definitive conclusions regarding these potential contributing factors.

Nevertheless, treatment duration is unlikely to be the main driver of this discrepancy. The median duration of treatment in our series (7.9 months) was comparable to that reported in DESTINY-Breast04 (8.2 months in the overall population), where discontinuations unrelated to disease progression still accounted for approximately 30% of cases [7].

Taken together, these observations indicate that differences in treatment exposure alone are unlikely to fully explain the lower proportion of discontinuations unrelated to disease progression observed in our cohort. Moreover, the differences observed between HER2-positive and HER2-low tumors in terms of treatment duration and response should also be interpreted in light of the different treatment histories of these populations. Patients with HER2-low tumors were more heavily pretreated than those with HER2-positive disease, with 72% receiving T-DXd in the fourth or later line compared with 34.8% of patients with HER2-positive tumors. This difference in treatment line is likely to have influenced both treatment duration and response, and therefore the observed differences cannot be attributed to HER2 status alone. The small sample size and retrospective nature of the study further limit the ability to disentangle the relative contribution of HER2 status, treatment line, and other baseline prognostic factors to these outcomes.

Discontinuations due to adverse events were numerically less frequent in our series (14%) compared with clinical trials, where rates ranged from approximately 18% to 35% (Supplementary Table S1). Differences in toxicity prevention and management strategies between our practice and the heterogeneous setting of multicentre trials may have contributed to this observation, although this hypothesis cannot be formally tested within the present study.

For example, dose reductions due to nausea and vomiting were less frequent in our series than in clinical trials (3% vs. approximately 6%), possibly reflecting the systematic use of antiemetic prophylaxis at our institution [12], which may have improved tolerability and treatment adherence, although a causal relationship with treatment adherence cannot be established from our data. Indeed, early T-DXd trial protocols did not include specific recommendations for antiemetic prophylaxis. However, our experience, together with data from randomized studies [13,14], supports the use of a three-drug antiemetic regimen, in line with recent antiemetic guidelines for T-DXd [15,16]. Importantly, differences in treatment duration are unlikely to fully account for this observation, as the patient-perceived burden of nausea and vomiting is greatest during the initial treatment cycles, after which their severity usually decreases and stabilizes over time [17,18]. Notably, no patient discontinued T-DXd due to nausea and vomiting in our study. Although our findings do not allow conclusions regarding causality, they support the importance of structured supportive care, including appropriate antiemetic prophylaxis, patient education, and early toxicity management, which warrant further evaluation in larger, multicenter cohorts to better characterize their potential impact on treatment exposure.

Discontinuations due to adverse events were largely driven by ILD, which accounted for approximately 11% of all treatment discontinuations, a proportion consistent with that reported in clinical trials (Supplementary Table S1). ILD is a well-recognized toxicity associated with T-DXd and is typically low grade (grade ≤ 2) in most patients. In our cohort, however, 50% of ILD events were grade ≥3. This finding should be interpreted taking into account that the present analysis included only patients who permanently discontinued treatment, inherently enriching the cohort for higher-grade events. The overall incidence of ILD observed in our cohort was comparable to that reported in clinical trials and may reflect the systematic and stringent monitoring adopted in our clinical practice. Despite this, three fatal ILD events occurred. One patient developed ILD very early, approximately 8 weeks after treatment initiation; she was a 52-year-old woman with a high burden of pulmonary metastatic disease and moderate renal impairment, both recognized risk factors for ILD [19]. The other two patients were receiving concomitant corticosteroid therapy for brain metastases, and one of them developed a concurrent opportunistic infection. Due to logistical constraints, these cases were managed outside our institution. Taken together, these observations further underscore the importance of proactive surveillance and timely management of ILD in patients receiving T-DXd, reinforcing current recommendations advocating systematic radiological monitoring, prompt diagnostic evaluation of new respiratory symptoms, and early multidisciplinary management throughout treatment.

Patients who discontinued T-DXd for reasons other than disease progression had a longer DoT than those who discontinued due to progressive disease. The substantial and durable efficacy of T-DXd observed in both clinical trials and real-world settings has prompted interest in treatment optimization strategies. In particular, maintenance approaches following an induction phase with T-DXd are currently under prospective evaluation and may represent one potential strategy to prolong treatment benefit while limiting cumulative toxicity in selected patients (e.g., NCT06172127).

Several real-world studies have reported on the efficacy and safety of T-DXd across different clinical settings [20,21,22,23,24,25,26,27,28,29,30]. However, most have primarily focused on treatment activity and safety outcomes, whereas detailed characterization of treatment discontinuation patterns, treatment exposure, dose modifications, and post-discontinuation therapeutic trajectories has remained limited. To the best of our knowledge, this is the first real-world study specifically focused on these clinically relevant aspects of T-DXd management.

The real-world design reflects routine clinical practice, including patient heterogeneity and treatment decisions outside the constraints of clinical trial protocols. In addition, the monocentric design allowed for consistent treatment management and toxicity monitoring, strengthening the internal coherence of the dataset. The detailed documentation of discontinuation reasons, dose modifications, and subsequent therapies provides clinically meaningful insight into treatment trajectories following T-DXd.

This study has limitations. Its retrospective design precluded full control over all potential confounding variables that may have influenced treatment duration and discontinuation patterns. In addition, adverse events were captured through retrospective chart review, which may have resulted in underreporting of lower-grade toxicities. Moreover, the retrospective nature of the analysis does not allow us to definitively identify which factors most strongly contributed to treatment adherence. Finally, as a single-center experience, the generalizability of our findings may be limited.

## 5. Conclusions

In this real-world cohort of patients with metastatic breast cancer treated with T-DXd, most permanent discontinuations were driven by disease progression, whereas adverse event-related discontinuations were less frequent and mainly attributable to ILD. Discontinuations due to other toxicities or patient choice were uncommon and occurred less often than reported in clinical trials. Overall, the relatively low proportion of discontinuations unrelated to disease progression observed in our series may reflect differences between routine clinical practice and clinical trial settings, although the factors underlying this observation cannot be determined from our retrospective analysis. Several factors, including toxicity management, may influence treatment persistence and warrant further evaluation in larger, multicenter cohorts.

## Figures and Tables

**Table 1:** Patient Baseline Characteristics.

Characteristics	Overall *n* = 93 (%)	HER2+ *n* = 66 (%)	HER2-Low *n* = 25 (%)	HER2 0 *n* = 2 (%)
Age				
Range	24; 85	24; 85	35;79	34; 48
Mean	57.1	56.8	59.0	41
Sex				
Female	93 (100)	66 (100)	25 (100)	2 (100)
ECOG PS				
0	61 (65.6)	46 (69.7)	14 (56)	1 (50)
1	30 (32.3)	20 (30.3)	9 (36)	1 (50)
≥2	2 (2.1)	0	2 (8)	0
N° of prior lines for metastatic disease				
Range	0; 13	0; 13	1; 12	2; 5
0	5 (5.4)	5 (7.6)	0	0
1	17 (18.3)	14 (21.2)	3 (12)	0
2	29 (31.2)	24 (36.4)	4 (16)	1 (50)
≥3	42 (45.1)	23 (34.8)	18 (72)	1 (50)
Visceral metastases				
no	23 (24.7)	19 (28.8)	4 (16)	0
yes	70 (75.3)	47 (71.2)	21 (84)	2 (100)
Brain metastases				
no	65 (69.9)	47 (71.2)	17 (68)	1 (50)
yes	28 (30.1)	19 (28.8)	8 (32)	1 (50)

## Data Availability

The datasets generated during and/or analysed during the current study are not publicly available but are available from the corresponding author on reasonable request.

## Supplementary Materials

The following supporting information can be downloaded at: https://www.mdpi.com/article/10.3390/cancers18172882/s1, Table S1: Patterns of treatment discontinuation in pivotal trials of trastuzumab deruxtecan in metastatic breast cancer.
