# Supplementary material for: Low Rates of Discontinuation Unrelated to Disease Progression with Trastuzumab Deruxtecan in Metastatic Breast Cancer: A Real-World Study
Source: Cancers (Basel). 2026 Sep 6;18(17):2882. doi: 10.3390/cancers18172882 (PMC13564716; doi:10.3390/cancers18172882)
Supplement: Supplementary file 1 [file cancers-18-02882-s001.zip › cancers-4510356-supplementary.pdf]

Supplementary Table S1. Patterns of treatment discontinuation in pivotal trials of trastuzumab deruxtecan in metastatic breast cancer

Trial	DESTINY Breast02	DESTINY Breast03	DESTINY Breast04	DESTINY Breast06	DESTINY Breast09	DESTINY Breast12†
No. of Pts treated	404 (of 406)	257 (of 261)	371 (of 373)	434 (of 436)	380 (of 383)	263
Median follow-up (mo)	30.2	43.0	32.0	18.2	29.2	15.4
Duration of treatment (mo)	11.3	18.2	8.2	11.0	20.0 (T-DXd+P: 21.7)	11.5
Total discontinuation (%)	86.4 (100)	80.5 (100)	95.4 (100)	79.5 (100)	59.7 (100)	55.1 (100)
PD (%)	47.8 (55.3)	41.6 (51.7)	69.0 (72.3)	57.1 (71.8)	21.3 (35.7)	30.8 (55.9)
AE (%)<br>ILD/pneumonitis	20.3 (23.5)<br>9.4 (10.9)	23.7 (29.4)<br>12 (14.9)	16.7 (17.5)<br>8.2 (8.6)	14.1 (17.7)<br>8.8 (11.1)	20.8 (34.8)<br>NA	11.8 (21.4)<br>10.3 (18.7)
Patient decision (%)	9.2 (10.6)	8.6 (10.7)	3.8 (4.0)	4.4 (5.5)	11.1 (18.6)	3.4 (6.2)
Clinical progression (%)	6.4 (7.4)	1.9 (2.4)	N.A.	N.A.	N.A.	2.3 (4.2)
Death (%)	1.2 (1.4)	1.6 (2.0)	1.3 (1.4)	1.2 (1.5)	2.4 (4.0)	2.7 (4.9)
Other reasons*	1.5 (1.8)	3.1 (3.8)	4.6 (4.8)	2.7 (3.5)	4.1 (6.9)	4.1 (7.4)

*Other reasons include: physician decision, lost to follow-up, other unknown reasons
† data refer to patients with baseline BMs
